# Constrained surfaces: promising therapeutic targets for COVID-19 determined by systematically mutational analysis

**DOI:** 10.1038/s41392-021-00469-8

**Published:** 2021-01-29

**Authors:** Manni Wang, Tianxia Lan, Wei Wang

**Affiliations:** Laboratory of Aging Research and Cancer Drug Targets, State Key Laboratory of Biotherapy and Cancer Center, National Clinical Research Center for Geriatrics, West China Hospital, Sichuan University, No. 17, Block 3, Renmin Road South, Chengdu, Sichuan 610041 P.R. China

**Keywords:** Infectious diseases, Bioinformatics, Target identification

A recent study published in *Cell* by Tyler N. Starr et al.^1^ presented a quantitative deep mutational scanning platform that allowed for the characterization of potential amino acid (AA) mutations to the SARS-CoV-2 receptor binding domain (RBD). The mutation-phenotype maps provided in this work determined the influence of RBD expression and binding affinity to human cell-surface protein angiotensin-converting enzyme 2 (ACE2) induced by fully random substitution for each AA of RBD. This work identified pivotal positions for virus binding and entry into cells, facilitating the development of countermeasures for COVID-19, such as vaccines.

Due to the ongoing COVID-19 pandemic, SARS-CoV-2 and its RBD-based vaccine approach, have received considerable research attention^2^. The RBD of spike protein from SARS-CoV-2 not only binds to its receptor ACE2 with high affinity allowing for viral entry^3^ but also functions as the target of multiple known antibodies to SARS-CoV-2. Despite recent research efforts on spike RBD mutations, it remains unclear whether mutations can affect biochemical phenotypes of RBD and thus yield resistance to neutralizing antibodies.

The team proposed a yeast-based platform for the systematic exhibition of RBD variants, which enabled the comprehensive analysis of SARS-CoV-2 RBD mutations. To improve the efficiency of deep mutational scanning, a specific 16-nucleotide barcode was respectively appended to each variant, which allowed for the high-throughput sequencing into the rapid linkage between mutations and phenotypes and the subsequent visualization of how RBD mutations impact expressions and their ACE2-binding affinity. Through the sequence-to-phenotype heatmaps, a number of conserved regions in the RBD sequence were identified. Moreover, it is noteworthy that several RBD mutants were found to enhance expression or ACE2-binding affinity, which would possibly be selected over the course of evolution. In general, such a visualization approach not only provides important clues for vaccine design but also facilitates the identification of potentially infectivity-enhancing mutations that might be surveilled with caution.

Next, they integrated the mutation-to-phenotype map with the SARS-CoV-2 RBD crystal structure and characterized several mutational constraints on RBD. Interestingly, constraints on ACE2-binding predominantly locate on the receptor-binding motif (RBM) subdomain, whereas constraints on expression commonly focus on the core-RBD subdomain. This observation accords with an earlier report^4^, suggesting RBM as a functional region of RBD for ACE2-binding. From the three-dimensional mapping of constraints, the researchers also found that expression-enhancing mutations could either positively or negatively affect ACE2-affinity based on their positions. For instance, a higher level of RBD expression always correlated with higher ACE2-binding affinity, since stable protein folding is an essential prerequisite for tight binding. On the other hand, although some variants displayed a higher expression level of RBD, their binding affinity to ACE2 was reduced, which might be attributed to the steric clashes with the receptor.

**Fig. 1:**
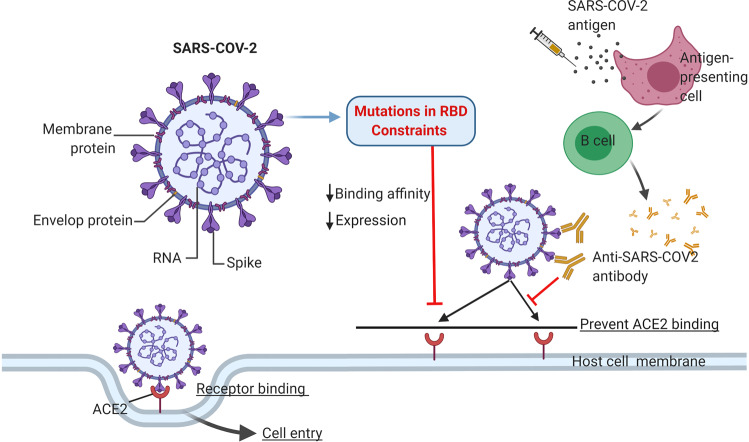
Prevention of SARS-Cov-2 viral-escape by targeting RBD constraints. The figure is created by Biorender

Furthermore, the team sought to establish the correlation between phenotypes of different sarbecovirus clades and the mutational tolerance of a single residual. Notably, some residues in ACE2 contact positions with SARS-CoV-2 RBD that were annotated as crucial factors for SARS-CoV-1 were found to be tolerable to mutations, indicating remarkable degeneracy at such sites. Moreover, the deep mutational scanning approach provided in the study would be more applicable for the prediction of the ACE2-binding ability of viruses with fewer mutations to the SARS-CoV-2 clade. For instance, the predicted increase in ACE-2 binding affinity of GD-Pangolin was in line with the affinity-enhancing mutations observed in its RBD sequence, which accords with a previous report^5^. In addition, the map was used to assess the mutation tolerance of constraints in RBD-binding antibody epitopes and demonstrated that epitopes of currently known antibodies were less constrained than the ACE2 interface itself. Since viral escape is less likely to occur on RBD surfaces carrying highly constrained epitopes, this finding encourages the design of antibodies with further targeting of these constraints (Fig. 1). According to mutations identified in pandemic isolates, affinity-enhancing mutations had so far not been naturally selected.

Previously developed strategies have provided structure-based intervention that prevents ACE2 recognition by SARS-CoV-2^4^. The high-throughput map provided in the study evaluated phenotypic consequences of mutations and their impacts on ACE2 binding in SARS-CoV-2 clade. Although previous studies have identified reduced infectivity in some viral strains following amino acid changes at RBD, the natural variants of RBD should be closely monitored due to potential resistance to neutralizing antibodies. Further studies are warranted to define the evolution of RBD that enables sarbecoviruses to be efficiently introduced to the human population.
